# Salvage of preputial flap necrosis in penile glans melanosis using Integra dermal regeneration template and split-thickness skin graft: A case report

**DOI:** 10.1097/MD.0000000000048843

**Published:** 2026-05-15

**Authors:** Wenhua Wang, Yang Feng Lou, Long Fei Yang, Peng Zhou

**Affiliations:** a Department of General Surgery, Hangzhou Third People’s Hospital, Hangzhou, China; b Department of Urology, Hangzhou Third People’s Hospital, Hangzhou, China.

**Keywords:** dermal regeneration template, flap necrosis, Integra, negative-pressure wound therapy, penile glans melanosis, preputial flap, psychosexual outcome, split-thickness skin graft

## Abstract

**Rationale:**

Penile glans melanosis is a benign but psychologically distressing condition. While surgical excision with flap reconstruction is a standard treatment for extensive lesions, total flap necrosis presents a significant reconstructive challenge. This report describes a successful salvage strategy using a dermal regeneration template (Integra) and split-thickness skin grafting (STSG) after the failure of a primary preputial flap.

**Patient concerns:**

A 24-year-old male with a 2.0 × 1.5 cm benign melanosis of the glans penis underwent complete excision and reconstruction with a pedicled inner preputial flap. However, at the 3-week follow-up, the patient presented with complete flap necrosis, necessitating further intervention.

**Diagnoses:**

Postoperative complete necrosis of the pedicled inner preputial flap following excision of glans melanosis.

**Interventions:**

Following thorough debridement of the necrotic tissue, a staged salvage procedure was initiated. First, a single-layer Integra Dermal Regeneration Template was applied to the wound bed and managed with continuous negative-pressure wound therapy at −125 mm Hg for 3 weeks to promote neodermis formation. Subsequently, a 0.3-mm meshed STSG harvested from the right thigh was transplanted onto the matured neodermis and secured with negative-pressure wound therapy for 5 days.

**Outcomes:**

Complete epithelialization was achieved 6 months postoperatively, resulting in a smooth contour and minimal scarring. At the 24-month follow-up, the patient reported preserved erogenous sensation (2-point discrimination of 5 mm) and improved psychosexual satisfaction, with IIEF-15 scores increasing from 5/10 to 8/10. No recurrence or contracture was observed.

**Lessons:**

Staged reconstruction using a dermal regeneration template followed by STSG is a reliable and effective salvage technique for flap failure in glans surgery. This approach avoids the complications of secondary flap harvesting while ensuring excellent functional, sensory, and cosmetic outcomes, making it a valuable tool for complex penile surface reconstruction.

## 1. Introduction

Penile glans melanosis is a benign hyperpigmentation caused by increased melanin deposition in the basal epidermis without cellular atypia.^[1]^ Although it carries no malignant potential, extensive involvement often leads to significant anxiety and psychosexual impairment.^[2]^ nonsurgical treatments such as laser therapy are associated with high recurrence rates, particularly in darker skin types.^[3]^ For large lesions, complete excision followed by reconstruction is the most durable option.^[4]^ Pedicled preputial flaps provide thin, well-vascularized, sensate tissue ideally suited for glans resurfacing.^[5]^ However, flap necrosis, although uncommon, can compromise outcome.^[6]^ When necrosis occurs, secondary reconstruction must preserve sensation and contour while minimizing contraction.^[4]^ To our knowledge, the use of a dermal regeneration template as salvage after failed preputial flap reconstruction for benign penile glans melanosis has not been previously reported. We describe successful two-stage salvage using single-layer Integra® followed by split-thickness skin grafting, resulting in excellent long-term cosmetic and functional restoration.^[7]^

## 2. Case presentation

**Figure 1.**
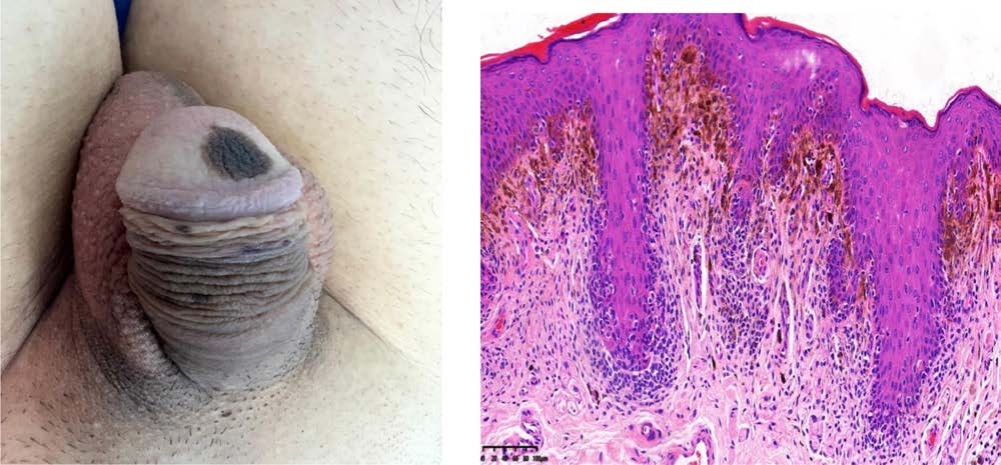
Preoperative clinical presentation of extensive penile glans melanosis, with histopathology confirming benign melanosis (H&E stain, ×40).

A 24-year-old healthy, uncircumcised man (BMI 22 kg/m^2^, nonsmoker, Fitzpatrick type III) presented with progressive brown-to-black macules involving approximately 40% of the glans surface over 3 years (Fig. 1). Routine blood tests were performed to assess overall health and rule out comorbidities; results are summarized in Table 1. Dermoscopy and 3-mm punch biopsy confirmed benign melanosis without atypia (Fig. 1). He reported severe psychosexual distress (IIEF-15 overall satisfaction domain score 5/10) and requested definitive surgical treatment.

**Table 1:** Clinical parameters of the patient.

Parameter	Value	Reference range
Hemoglobin (g/dL)	15.2	13.5–17.5
White blood cell count (×109/L)	7.8	4.0–11.0
Platelet count (×109/L)	250	150–450
Erythrocyte sedimentation rate (mm/h)	16	0–20
C-reactive protein (mg/L)	4.5	<10.0
Fasting blood glucose (mmol/L)	5.6	3.9–6.1
Creatinine (μmol/L)	88	60–110
Alanine aminotransferase (U/L)	27	7–56
Aspartate aminotransferase (U/L)	26	8–48
HIV serology	Negative	Negative

**Figure 2.**
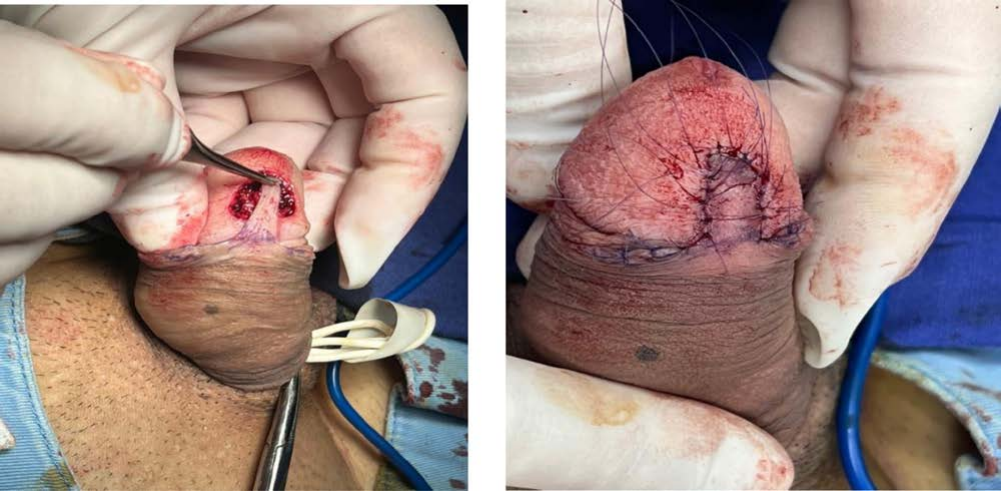
Post-excisional defect demonstrating histologically clear margins, with immediate postoperative appearance following preputial flap inset and reconstruction.

Under spinal anesthesia with penile tourniquet (250 mm Hg, 18 min), a 2.0 × 1.5 cm fusiform excision was performed with frozen-section clearance (Fig. 2).^[8]^ A pedicled inner preputial flap was harvested, rotated without tension, and inset with 6-0 polyglactin sutures (Fig. 2).^[5]^ Oral cefuroxime was given for 7 days.

**Figure 3.**
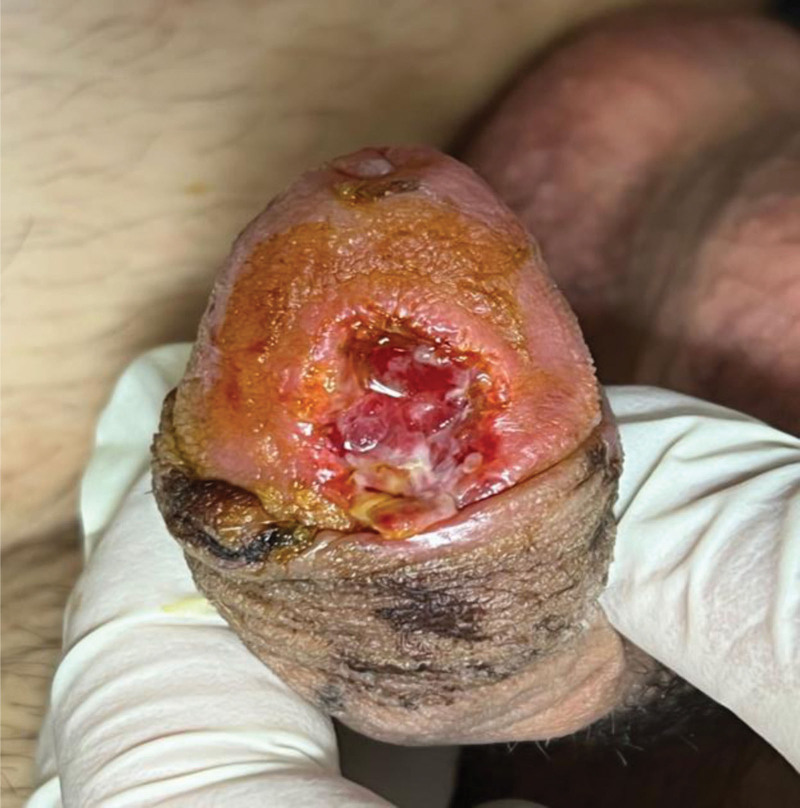
Progressive flap ischemia with complete necrosis by the 3-week postoperative follow-up.

**Figure 4.**
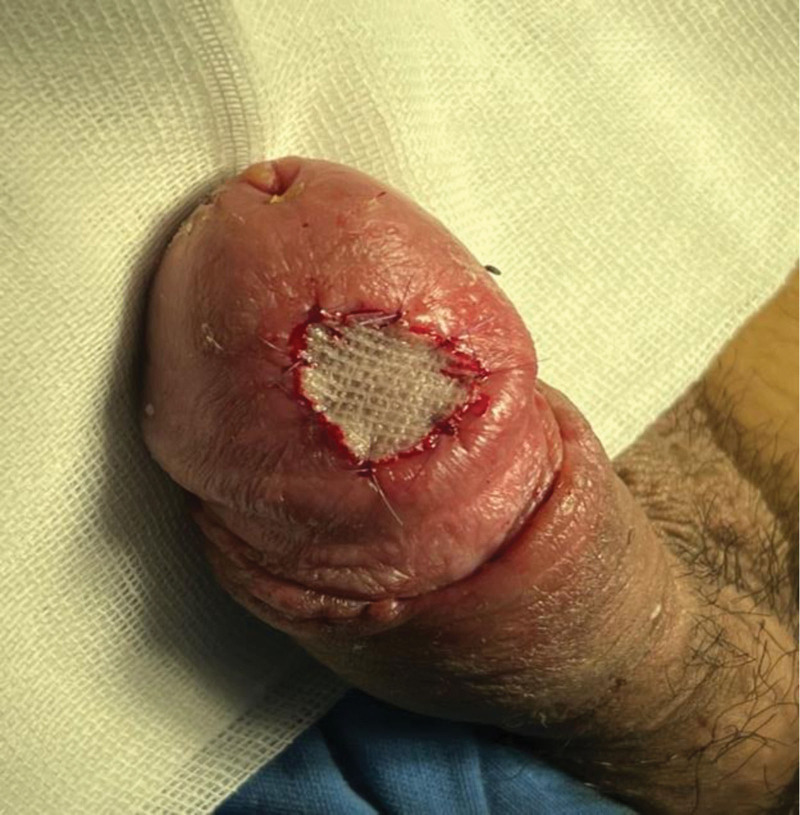
Wound coverage was achieved using single-layer Integra® Dermal Regeneration Template (Integra LifeSciences, Plainsboro, NJ, USA).

**Figure 5.**
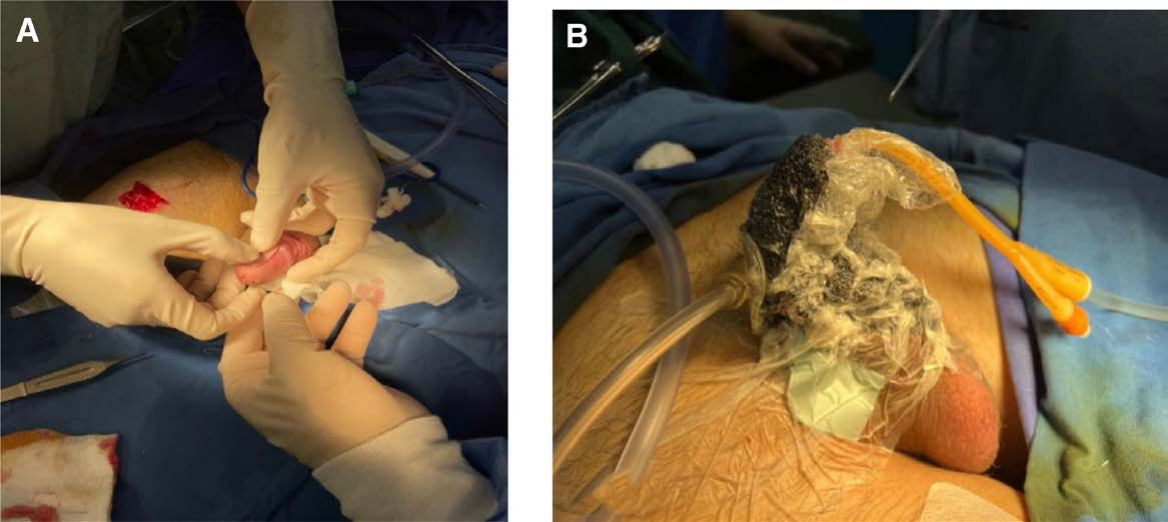
A. Split-thickness skin grafting was performed over the matured neodermis. (B) Negative-pressure wound therapy (NPWT) was applied over the Integra® template.

During the early postoperative period, progressive ischemia and partial necrosis were noted, which progressed to complete flap necrosis by postoperative week 3 (Fig. 3). The patient did not attend follow-up between postoperative week 1 and week 3. Under local anesthesia (10 mL 1% lidocaine with 1:200,000 adrenaline) in the operating theater, necrotic tissue was sharply debrided and the pedicle ligated. Single-layer Integra® Dermal Regeneration Template was hydrated, trimmed, applied, and secured with staples and continuous negative-pressure wound therapy at -125 mm Hg (Fig. 3). After confirmed neodermis formation 3 weeks later (week 6), a 0.3-mm meshed split-thickness skin graft (1.5 × 1.5 cm) from the right medial thigh was placed and fixed with negative-pressure therapy (−125 mm Hg) for 5 days (Figs. 4 and 5). The smaller graft size reflected mild wound contraction during the neodermis maturation phase under negative-pressure wound therapy.

**Figure 6.**
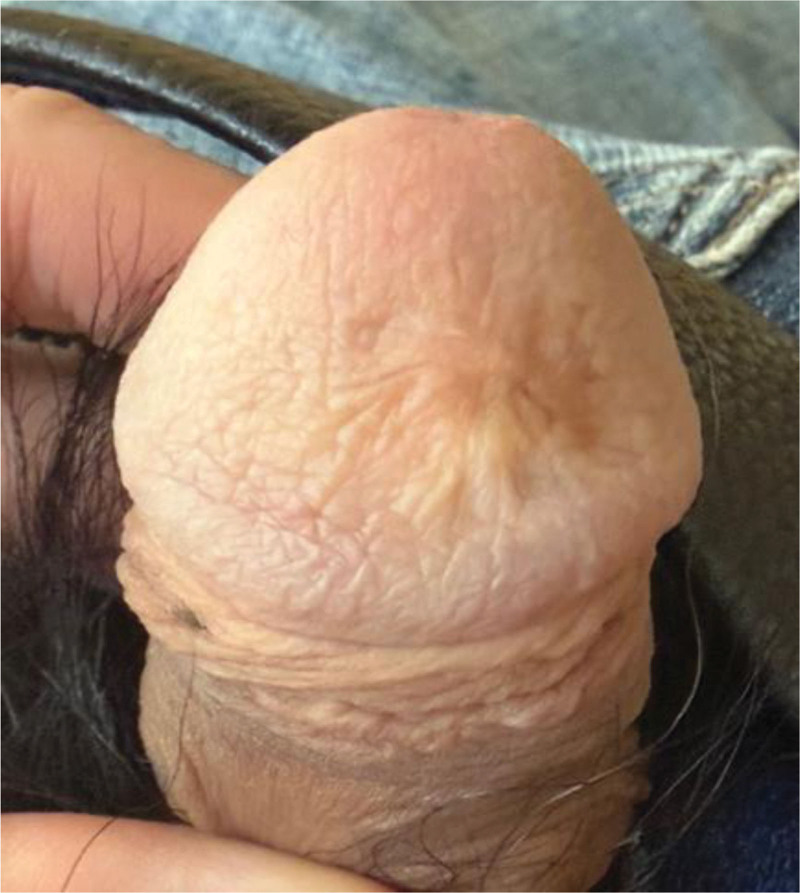
Clinical appearance and scar maturation at 24 months postoperatively, demonstrating excellent contour restoration and near-normal skin texture.

At 6 months, the glans was fully epithelialized with a smooth contour and 1-mm scar. Two-point discrimination was 5 mm. IIEF-15 scores are shown in Table 2. At 24-month clinic follow-up, the patient completed the IIEF-15 questionnaire again, and the IIEF-15 overall satisfaction domain score had improved to 8/10; no recurrence, dyschromia, or contracture was noted (Fig. 6). A clear clinical timeline was as follows: initial surgery (week 0), progressive flap ischemia evolving to complete necrosis by week 3, debridement and Integra application at week 3, split-thickness skin grafting at week 6, and complete epithelialization at 6 months.

**Table 2:** Pre- and postoperative international index of erectile function-15 (IIEF-15) scores.

Domain	Preoperative Score	Postoperative Score (6 months)	Postoperative Score (24 months)
Erectile Function	22/30	24/30	25/30
Orgasmic Function	8/10	9/10	9/10
Sexual Desire	7/10	8/10	8/10
Intercourse Satisfaction	10/15	12/15	12/15
Overall Satisfaction	5/10	7/10	8/10

## 3. Discussion

This case demonstrates that complete preputial flap necrosis after glans resurfacing for benign melanosis can be successfully salvaged using a two-stage approach with Integra® and thin split-thickness skin grafting. The silicone layer of Integra provides temporary watertight coverage while the collagen-glycosaminoglycan matrix serves as a scaffold for dermal regeneration, resulting in reduced wound contraction and improved pliability compared with direct skin grafting alone.^[9]^ Negative-pressure wound therapy (−125 mm Hg) further enhances neovascularization, decreases bacterial load, and increases graft take rate to nearly 100% in genital reconstruction.^[7]^

Integra has been widely adopted in oncologic penile reconstruction, particularly after partial penectomy or glansectomy for squamous cell carcinoma, where large skin defects and adjuvant radiotherapy increase the risk of wound breakdown.^[10]^ Multiple series have confirmed its reliability in tumor-related settings, with satisfactory cosmetic and functional outcomes even in irradiated fields.^[10,11]^ However, to our knowledge, the present report is the first documented use of Integra as a salvage tool in a non-oncologic penile indication – specifically, after failed preputial flap reconstruction for benign glans melanosis. The absence of malignancy, previous radiation, or chronic inflammation in this young patient resulted in an even more favorable healing environment, with minimal scar contracture and preservation of erogenous sensation (two-point discrimination 5 mm) at 24 months.

This strategy may be preferable to immediate re-flapping in the salvage setting because it avoids additional donor-site morbidity, eliminates the risk of recurrent pedicle ischemia, and provides a robust neodermis that closely mimics native glans tissue. Notably, compared with immediate re-flapping, the staged Integra-based approach offers several advantages in the salvage setting, including avoidance of further vascular compromise, reduced donor-site morbidity, and formation of a pliable neodermis that closely mimics native glans tissue. This may be particularly advantageous in young patients with benign disease, where preservation of sensation and cosmetic outcomes are of paramount importance.

Given the excellent long-term cosmetic contour, restored erectile function, and significant improvement in quality of life observed here, staged Integra-based reconstruction should be considered a valuable option when primary flap failure occurs in benign glans resurfacing procedures.

## 4. Conclusions

Staged reconstruction using a dermal regeneration template followed by thin split-thickness grafting is an effective salvage strategy after preputial flap necrosis in benign glans resurfacing, yielding excellent cosmetic, sensory, and functional outcomes.

## Author contributions

**Conceptualization:** Peng Zhou.

**Investigation:** Wenhua Wang, Yang Feng Lou.

**Data curation:** Yang Feng Lou.

**Formal analysis:** LongFei Yang.

**Supervision:** Peng Zhou.

**Writing – original draft:** Wenhua Wang.

**Writing – review & editing:** Peng Zhou.
